# Why the 2022 Po River drought is the worst in the past two centuries

**DOI:** 10.1126/sciadv.adg8304

**Published:** 2023-08-09

**Authors:** Alberto Montanari, Hung Nguyen, Sara Rubinetti, Serena Ceola, Stefano Galelli, Angelo Rubino, Davide Zanchettin

**Affiliations:** ^1^ Department of Civil, Chemical, Environmental and Materials Engineering (DICAM), Alma Mater Studiorum Università di Bologna, Bologna, Italy.; ^2^ Lamont-Doherty Earth Observatory, Columbia University, Palisades, NY, USA.; ^3^ Alfred Wegener Institute, Helmholtz Centre for Polar and Marine Research, List/Sylt, Germany.; ^4^ Pillar of Engineering Systems and Design, Singapore University of Technology and Design, Singapore, Singapore.; ^5^ Department of Environmental Sciences, Informatics and Statistics, Ca’ Foscari University of Venice, Venice, Italy.

## Abstract

The causes of recent hydrological droughts and their future evolution under a changing climate are still poorly understood. Banking on a 216-year river flow time series at the Po River outlet, we show that the 2022 hydrological drought is the worst event (30% lower than the second worst, with a six-century return period), part of an increasing trend in severe drought occurrence. The decline in summer river flows (−4.14 cubic meters per second per year), which is more relevant than the precipitation decline, is attributed to a combination of changes in the precipitation regime, resulting in a decline of snow fraction (−0.6% per year) and snowmelt (−0.18 millimeters per day per year), and to increasing evaporation rate (+0.013 cubic kilometers per year) and irrigated areas (100% increment from 1900). Our study presents a compelling case where the hydrological impact of climate change is exacerbated by local changes in hydrologic seasonality and water use.

## Teaser

Changes in precipitation and evaporation regimes, river flow seasonality and water use increase drought severity in the Po River.

## INTRODUCTION

During the first 7 months of 2022, a severe meteorological drought occurred over a large part of the European continent, associated with a persistent anomalous anticyclonic circulation over its northwestern portion (*1*). In particular, an extraordinary scarcity of precipitation hit Northern Italy, contributing to a prolonged hydrological drought that threatened water resources security and riverine ecosystems. The Po River, the longest water course in Italy, reached critically low levels, cutting the availability of irrigation water and leading to record-breaking seawater intrusion (*2*). The severity of the 2022 Po River drought and its potential long-lasting impacts were amply covered by the media worldwide as the Italian government declared a state of emergency in the five administrative regions of the Po Valley (*3*). Several towns had to ration water amidst the drought, and extraordinary measures were taken to support farmers, as the Po Valley supplies around 40% of the country’s food demand. The drought also affected the productivity of both hydropower and thermoelectric stations (*4*), a reason of major concern amid the ongoing global energy crisis. The severity of this event concerns on the increasing frequency of hydrological droughts (*5*–*7*), their socioeconomic impact, and the future state of water resources (*8*).

Climate model projections for Europe indicate that meteorological droughts, due to lacking precipitation, will become increasingly more frequent and severe, especially in the second part of the 21st century (*9*). Looking at the Po Valley and, more broadly, at the Mediterranean area, projections up to 2100 from the climate models developed within the sixth phase of the Coupled Model Intercomparison Project suggest that drought frequency and intensity will increase with high confidence (*10*). However, it is not yet clear whether hydrometeorological observations display a trend in drought severity; the identification of a climate change signal is still limited to single meteorological events or subregional weather behaviors (*10*). By looking at historical data, various metrics of precipitation occurrence in Europe for the period 1850–2018 indicate a general absence of long-term trends (*11*). The problem of identifying and explaining long-term trends is even more challenging when looking at river flow and thus hydrological droughts, owing to the presence of local human influences such as land use change and water withdrawals (*8*, *12*). Moreover, the length of river flow time series is often limited to about 100 years at best (*13*, *14*). However, water management decisions and climate change adaptation measures would be best informed by a complete understanding of trends in river flow over prolonged periods of time (*15*, *16*). The 2022 Po River drought encapsulates all these matters: The cumulative precipitation from November 2021 to July 2022 over the Po River basin was not exceptionally low (*17*), therefore highlighting that additional drivers other than precipitation scarcity exacerbated the 2022 hydrological drought. The questions of interest are therefore the following: Is the 2022 Po River drought part of a long-term trend whereby droughts in Northern Italy are increasing in frequency and severity? And, if so, what are the causes of these trends?

Here, we answer these questions by analyzing the longest record of the Po River discharge, namely, a time series of monthly river flows at Pontelagoscuro (near the basin outlet), spanning from January 1807 to August 2022 (fig. S1). The time series combines an existing reconstruction (*18*, *19*) with recent data retrieved from the National Hydrographic Service (see Materials and Methods). We first focus on the spring–summer months by computing the annual values of the mean river flow for four aggregation windows: July (J), June–July (JJ), May–June–July (MJJ), and April–May–June–July (AMJJ), therefore obtaining 216-year long seasonal records. We then locate the position of the 2022 seasonal flow values in the ordered samples to assess the extraordinary severity of the recent drought. The characterization of the 2022 event is complemented by a drought frequency analysis carried out for the four aggregation windows. We then use quantile regression to analyze the temporal evolution of the seasonal mean flows and detect changes in seasonality as a possible driver. Last, we unfold the nature of these changes by analyzing trends in rainfall and snow regimes as well as the temporal evolution of evaporation, water abstractions, and irrigated areas.

We show that the magnitude of the 2022 drought is unprecedented in the past two centuries and that this event is part of a long-term trend characterized by an increase in the frequency and severity of droughts, particularly during the past decades. We also show that the key drivers are changes in river flow seasonality, likely caused by a shift from solid to liquid precipitation, earlier snowmelt, increasing evaporation, and increasing water abstractions during summer.

## RESULTS

### A record-breaking drought

**Fig. 1.**
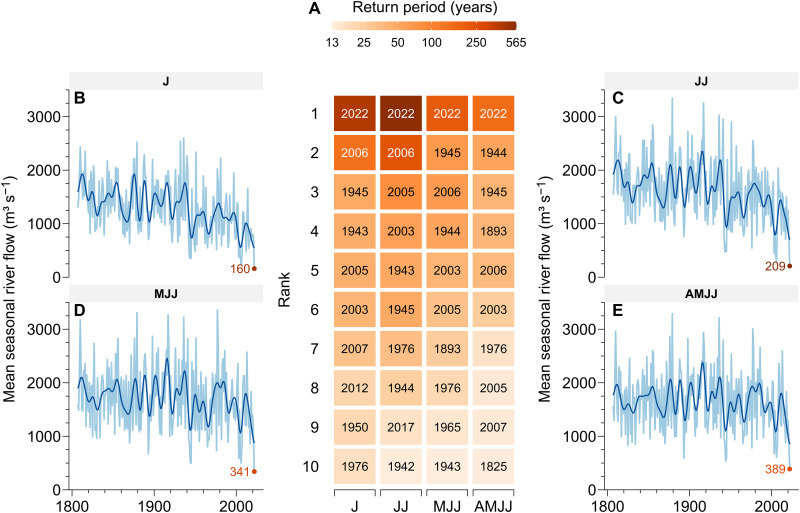
Evidence of the record-breaking 2022 Po River drought. (**A**) Ten worst droughts according to each aggregation window: J, JJ, MJJ, and AMJJ. The numbers denote the years of drought, and the color shows the corresponding return period. (**B** to **E**) Mean river flow during the observation period according to each aggregation window. The light blue lines show the seasonal mean river flow, while the dark blue lines indicate the 10-year centered moving average. The river flows recorded in summer 2022 are reported in shades of red according to their return periods.

Our analysis shows that the 2022 drought is a record-breaking event: No matter the aggregation window considered, the mean river flow observed in summer 2022 is, by far, the worst in the past two centuries (Fig. 1 and fig. S2). The second worst years are 2006 for the periods J and JJ and 1945 and 1944 for the periods MJJ and AMJJ, respectively (Fig. 1A). However, the 2022 records are about 30% lower of the respective second worst, thus marking the exceptionality of the 2022 event. We estimate the return period of seasonal river flows (under the assumption of stationarity) by fitting the observed records with the Weibull probability distribution (see Materials and Methods), which is widely used for drought frequency analysis (Fig. 1A and table S1) (*20*). The return period for JJ mean river flow approaches six centuries. By contrast, cumulative precipitation over the Po River basin from November 2021 to July 2022 amounts to 639 mm, corresponding to a low value with a return period of less than 50 years (see Materials and Methods). Therefore, the severity of the 2022 drought is much stronger than what one would expect from a 1-in-216-year extreme event and from the cumulative precipitation during the preceding season. Figure 1 also shows that the frequency of droughts has markedly increased after 2000: For the window of July, 6 of the 10 worst droughts since 1807 have occurred after 2000.

### Trend in drought severity

**Fig. 2.**
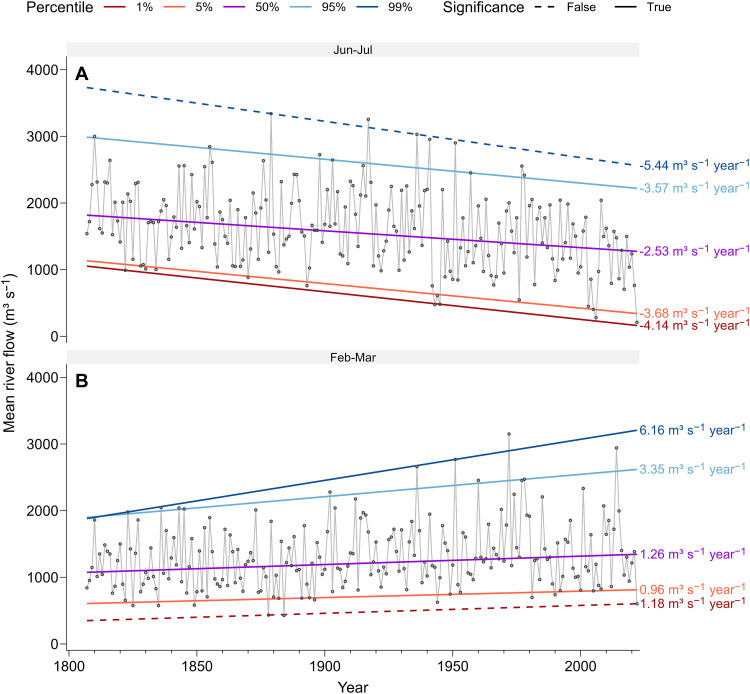
Seasonal Po River flow and quantile regression. Mean river flow in June–July (**A**) and February–March (**B**). In both panels, the linear trend in the 1st, 5th, 50th (median), 95th, and 99th percentile river flow is also shown. Trends are based on a 10% significance level.

Figure 1 also reveals that the 2022 Po River drought appears not to be an isolated event but rather part of a declining trend in river flow, which is visible from the aggregated time series (Fig. 1, B to E), especially those of the J and JJ windows (Fig. 1, B and C). To quantify these trends and test their significance, we use quantile regression (*21*, *22*) to regress the 1st, 5th, 50th, 95th, and 99th percentiles of each river flow time series against time. We find that significant declining trends are prevalent in the four aggregation windows (table S2). Here, we focus our discussion on JJ, being the most important period for irrigation. As shown in Fig. 2A, significant declining trends (*P* < 0.1) are observed from the 1st to the 95th percentiles. Low flows (i.e., 1st and 5th percentiles) are of particular concern, because they have been declining at a higher rate (−4.14 and −3.68 m^3^ s^−1^ year^−1^, respectively). Within such a long-term context of multicentennial decrease in the distribution of JJ river flow, the declining trend appears exacerbated after 1940: The 95th and 99th percentiles decrease at a rate of −20.87 and −16.55 m^3^ s^−1^ year^−1^, respectively.

### Change in river flow seasonality

Having identified a decline in summer river flow, we place this decline in the context of other seasons. Keeping the focus on JJ, we apply quantile regression to all bimonthly windows (i.e., December–January, February–March, April–May, June–July, August–September, and October–November). Most notably, consistent increasing trends are detected in the 5th to 95th percentiles of February–March mean river flow (Fig. 2B and table S2; results for other seasons are shown in fig. S4). The steady decline of summer river flow, coupled with consistent increase in spring flow, suggests that a change in seasonality has been occurring during the past two centuries. Our analysis is complemented by a lowess smoothing interpolation of February–March and June–July river flows, shown in fig. S3.

### Change in precipitation

**Fig. 3.**
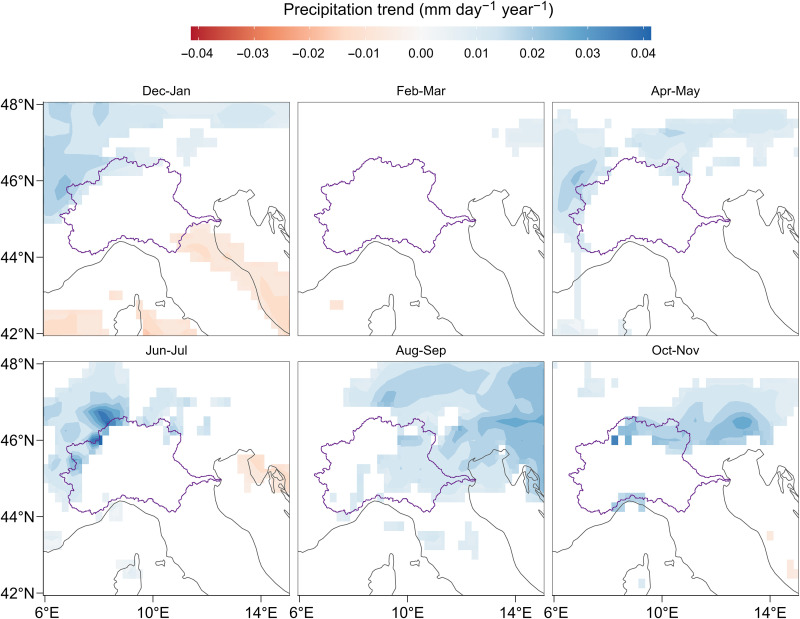
Change in Alpine precipitation regime. Precipitation trend estimation for the period 1940–2022 from ERA5 Reanalysis bimonthly data (*23*). Only significant trends (*P* < 0.1) are shown. The Po River basin boundary is shown in purple.

To determine whether the declining trend in river flow is caused by a corresponding decline in precipitation, we analyze the evolution of bimonthly cumulative precipitation over the Po River basin during the period 1940–2022 using the ECMWF Reanalysis v5 (ERA5) dataset (*23*). ERA5 is derived by combining model data with observations to obtain a spatially distributed representation of precipitation at a resolution of 0.25°, therefore allowing a detailed estimation of mean areal precipitation over large catchments. Our results show that no significant (*P* < 0.1) negative trend can be detected in precipitation over the Po River basin (Fig. 3) in any bimonthly window, thereby confirming previous analyses of long observational rainfall records in Northern Italy (*19*, *24*). Therefore, rainfall changes in individual months do not emerge as a major driver of the decreasing trend in river flow (*25*, *26*). Furthermore, we found that no significant trend can be detected in cumulative November–July precipitation for the periods 1940–2022, 1960–2022, 1980–2022, and 2000–2022 (fig. S5). Therefore, precipitation changes do not emerge as a notable driver of the decreasing trend in river flow.

### Change in snowmelt and solid precipitation regime

**Fig. 4.**
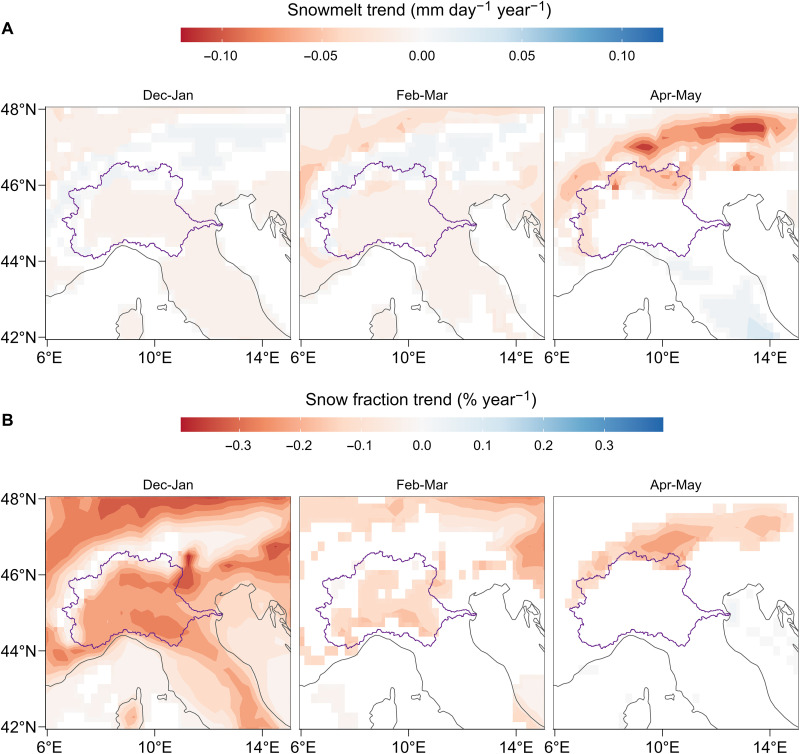
Change in Alpine snow regime. Snowmelt (**A**) and snow fraction (**B**) trend estimation for the period 1940–2022 from ERA5 Reanalysis bimonthly data (*23*). Snow fraction is calculated as the ratio between snowfall and total precipitation. Periods where trends are most pronounced are shown here (see figs. S6 and S7 for all periods). Only significant trends (*P* < 0.1) are shown. The Po River basin boundary is shown in purple.

Change in seasonality (Fig. 2) suggests that the form of precipitation (rain or snow), rather than its total amount, may explain the observed river flow trend. Global warming has led to changes in snow regimes (*9*, *27*–*29*), which, in turn, have shifted flood timing across Europe (*30*) and spring river flow timing in alpine rivers (*31*). Earlier snowmelt, induced by warming, has also led to both increases and decreases in flood magnitude (*32*). We thus hypothesize that the observed trends and changes in seasonality of the Po River flow may have been caused by changes in the snow regime, given the relevant contribution of snowmelt to it (*31*, *33*). To test this hypothesis, we look for trends in the ERA5 dataset (*23*) of bimonthly snowmelt and snow fraction (the ratio between snowfall and total precipitation) in the Alpine arc surrounding the Po River basin. Large and significant decreases in snowmelt (from −0.1 to −0.2 mm day^−1^ year^−1^; *P* < 0.1) are found around the headwaters of the Po River during spring. In colder months (December–March) we also observe a decline in snowmelt at lower elevations, nearer to the Po River Valley (Fig. 4A, first two panels, and fig. S6). Large and statistically significant downward trends in snow fraction (from −0.3 to −0.6% year^−1^; *P* < 0.1) are detected across the Alpine headwaters of the Po River from December to May (Fig. 4B and fig. S7). These trends are concomitant with increases in temperatures according to the ERA5 (*23*) and the Historical Instrumental Climatological Surface Time Series Of The Greater Alpine Region (HISTALP) (*34*, *35*) datasets (table S3). We conclude that the decline in snowmelt and snow fraction have very likely contributed to the reduction in the Po River flow in June and July. Warming temperatures have induced a prevailing liquid precipitation, rather than solid, which have reduced snowmelt processes during summer. Note that the above trends do not explain the early spring (February–March) increase in river flow (Fig. 2B). However, the statistically significant warming over the Po River basin in the months of December, January, and February (the latter is significant only for the ERA5 data; see table S3) supports the hypothesis that changes in snow fraction, although not statistically significant, may have contributed to the increase in early spring river flows.

### Change in evaporation

**Fig. 5.**
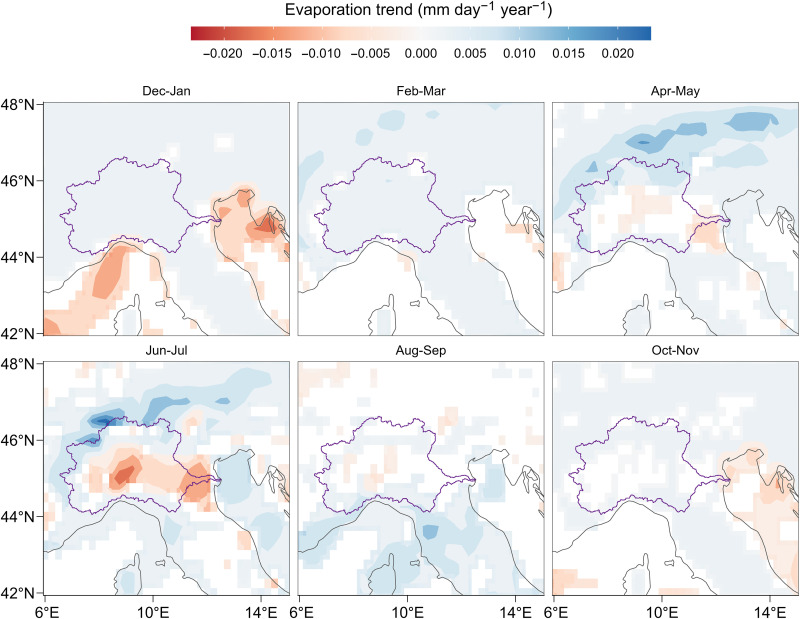
Change in total evaporation. Evaporation trend estimation for the period 1940–2022 from ERA5 Reanalysis bimonthly data (*23*). Only significant trends (*P* < 0.1) are shown. The Po River basin boundary is shown in purple.

A third driver may be the increased evaporation. Consistent with the analyses performed for the other drivers, we use the ERA5 dataset (*23*), which provides total evaporation estimates, including transpiration from vegetation. As shown in Fig. 5, the trends in bimonthly evaporation over the Po River basin are predominantly positive. This increase in total evaporation, particularly along the Alpine range, is of key relevance here, because evaporation over the Alps can be above average despite low precipitation, thereby amplifying the runoff deficit (*36*). Note the presence of a few areas with decreasing trends (for the months of June and July), which, however, do not largely affect the trend in the total annual evaporation across the Po River basin. Over the period 1940–2022, we found a statistically significant trend of +0.013 km^3^ year^−1^ (*P* = 0.01). This upward trend is exacerbated after 1980 (see fig. S8). Therefore, the increase in evaporation across the basin emerges as an additional, important, driver.

### Change in water withdrawals

A fourth driver may be represented by water abstraction, particularly due to surface water withdrawals for irrigation (*37*), which accounts for the majority (~75%) of total withdrawals from the Po River reach (*33*, *38*). Irrigation consortia have acknowledged increased water demands during spring and summer in recent times, mainly due to the anticipation of the irrigation season. To test the significance of this additional driver, we extract and analyze gridded data for the Po River basin from a global dataset of sectoral water use from 1970 to 2010 (*39*, *40*), which reveals negative, but nonsignificant, trends for irrigation water withdrawals during summer (table S4). However, such dataset may not consider local features in detail, and, therefore, we analyze the progress in the Po basin of the area equipped for irrigation (*37*, *41*, *42*), which has sizably increased over time, from 0.86 million ha in 1900 to 1.38 million ha in 1960, up to 1.63 million ha in 2015 (fig. S9). This pattern reflects the development of hydraulic infrastructures from 1940s to 1960s, followed by an overall plateau up to these days, which provides support to the hypothesis that irrigation has increased with a similar trend. The complementary analysis of other sectoral water uses reveals statistically significant increasing trends for domestic water withdrawals during summer (table S4). Overall, the above results provide support to the hypothesis that water withdrawals have significantly increased over time during the whole observation period.

## DISCUSSION

Our analysis reveals that the long-term decreasing trend in summer flows of the Po River, which steepened after 1940, is due to the superposition of several drivers. Among these, the reduction of the solid precipitation regime, the anticipation of snowmelt, and the raising total evaporation trace back to rising temperatures in the region after the Little Ice Age (*43*) and, more recently, under global warming. The temperature rise over the basin is particularly evident since the mid-20th century compared to the corresponding values over the past two centuries, which is reflected in particularly strong evaporation during the past 40 years and the months of June and July, thus confirming warming as one root of the observed long-term trend in summer flows of the Po River. A fourth driver is the expansion of irrigated areas, mainly occurring before the middle of the 20th century, which resulted in increased surface water abstractions, compounded by rising domestic water withdrawals. Water use has a markedly higher effect on trends in low flows than on high or average flows, thus explaining why the hydrological droughts in the Po River are often amplified with respect to the meteorological droughts. Our results confirm that complex interactions between several factors, including climate variability and change, land use and land cover change, and water management, trigger hydrological droughts (*8*). However, a rigorous quantification of the relative contribution of each of the above drivers to the increase of drought frequency and intensity would require now unavailable water abstraction data over the catchment and temporally extended spatially distributed climatic information. Furthermore, compounding feedbacks in the drought process limit an exhaustive attribution analysis (*8*). Still, we conclude that the combination of the above causes, together with a moderately lower than usual cumulative precipitation, caused in 2022 the worst hydrological drought ever observed since 1807, whose return period was estimated in more than four centuries for the summer flows. Looking forward, we expect that the ongoing global warming will exacerbate the trends analyzed here. In turn, their superposition is likely to worsen the severity and frequency of hydrological droughts, possibly exceeding the magnitude of the 2022 event. This calls for urgent search for solutions to mitigate the environmental and societal risks, thereby ensuring the sustainability of ecosystems and water resources (*44*).

## MATERIALS AND METHODS

### River flow time series

The Po River stage (i.e., water level) has been gauged in several locations since the onset of the 19th century (*18*). In particular, the river stage at Pontelagoscuro, conventionally considered the closure of the more than 70,000-km^2^ Po River basin, has been monitored since 1807. From 1917, river stages have been regularly converted to river flows by the National Hydrographic Service of Italy (*33*). The rating curve at Pontelagoscuro, estimated by the National Hydrographic Service of Italy in the 1920s (*45*), has been used to reconstruct the Po River monthly flows (*18*) from 1807 to 1916. The resulting time series obtained by merging the reconstruction with modern instrumental data (spanning the period from 1807 to today) has been used in several studies, including comparative assessments that support the robustness of the reconstruction (*15*, *19*, *46*, *47*). Here, we analyze the 216-year monthly record from January 1807 to August 2022 (fig. S1). Our dataset shows the effect of human impact, particularly on high river flows, where the development of the levee system along the Po River floodplains led to a reduction of storage capacity, which, in turn, caused an increase of the peak discharge.

Monthly precipitation (rain and snow), snow fraction, and evaporation data are retrieved from the ERA5 dataset (*23*), which consists of gridded data with a horizontal resolution of 0.25°. Monthly temperature data are retrieved from the ERA5 (*23*) and HISTALP (*34*, *35*) datasets. The HISTALP dataset consists of gridded data with a horizontal resolution of 5 × 5 min.

Monthly sectoral water withdrawals from 1971 to 2010 are retrieved from the global gridded monthly sectoral water use dataset (*39*, *40*), which is the first reconstructed global water use data product at subannual and gridded resolution (0.5°). Therein, water withdrawals are derived from different models and data sources, including Food and Agriculture Organization AQUASTAT and U.S. Geological Survey records.

### Return period analysis

To obtain an estimate of the probability of occurrence of the 2022 drought, we fit a Weibull distribution to the 216-year-long records of mean seasonal river flow. The analysis is carried out for four aggregation windows, namely, J, JJ, MJJ, and AMJJ, under the assumption of stationarity. We check the presence of dependence by computing the autocorrelation function of the seasonal data up to lag 24 (2 years). Autocorrelation coefficients are never statistically significant at the 95% confidence level for the JJ, MJJ, and AMJJ aggregation widows. A mild positive correlation is found for the J aggregation window, which we assume has a negligible effect when estimating the distribution parameters (*48*). The distribution provides a good fit to the data for all aggregation windows (fig. S2). The figure also shows that the 2022 drought marks the lowest point in all graphs. Results indicate that, in June and July 2022, the Po River was hit by a hydrological drought whose return period oscillates between four and six centuries (Fig. 1 and table S1). Note that these are the two most critical months concerning irrigation water supply. The return period of cumulative precipitation over the Po River basin from November 2021 to July 2022 is also computed. We assume stationarity and interpolate the empirical cumulative probability distribution function of the mean areal precipitation over the Po River basin derived from the ERA5 data (*23*), thus obtaining an estimate of 47 years.

### Trend estimation

The progress of mean monthly river flows at the closure of the Po River basin in Pontelagoscuro does not show any clear evidence of nonstationarity in the mean (fig. S1). To test for trends in the seasonal distribution of river flow, we use quantile regression (*21*) to regress different quantiles of the river flow distribution against time. Specifically, we use the 1st, 5th, 50th (median), 95th, and 99th percentiles to represent the distribution. To quantify the uncertainty in trend estimation, each quantile regression is bootstrapped 200 times (*49*).

### Climate data and trend analysis

Trend analysis for climate data is performed through a linear trend estimation.

### Water withdrawal data and trend analysis

Mann-Kendall test and Sen’s slope for water withdrawals are estimated to inspect temporal trends.
