# Supplementary material for: Why the 2022 Po River drought is the worst in the past two centuries
Source: Sci Adv. 2023 Aug 9;9(32):eadg8304. doi: 10.1126/sciadv.adg8304 (PMC10411875; doi:10.1126/sciadv.adg8304)
Supplement: Supplementary file 1 — Figs. S1 to S9 Tables S1 to S4 [file sciadv.adg8304_sm.pdf]

Science Advances
AAAS

Supplementary Materials for

Why the 2022 Po River drought is the worst in the past two centuries

Alberto Montanari *et al.*

Corresponding author: Alberto Montanari, alberto.montanari@unibo.it

Sci. Adv. **9**, eadg8304 (2023)
DOI: 10.1126/sciadv.adg8304

This PDF file includes:

Figs. S1 to S9
Tables S1 to S4

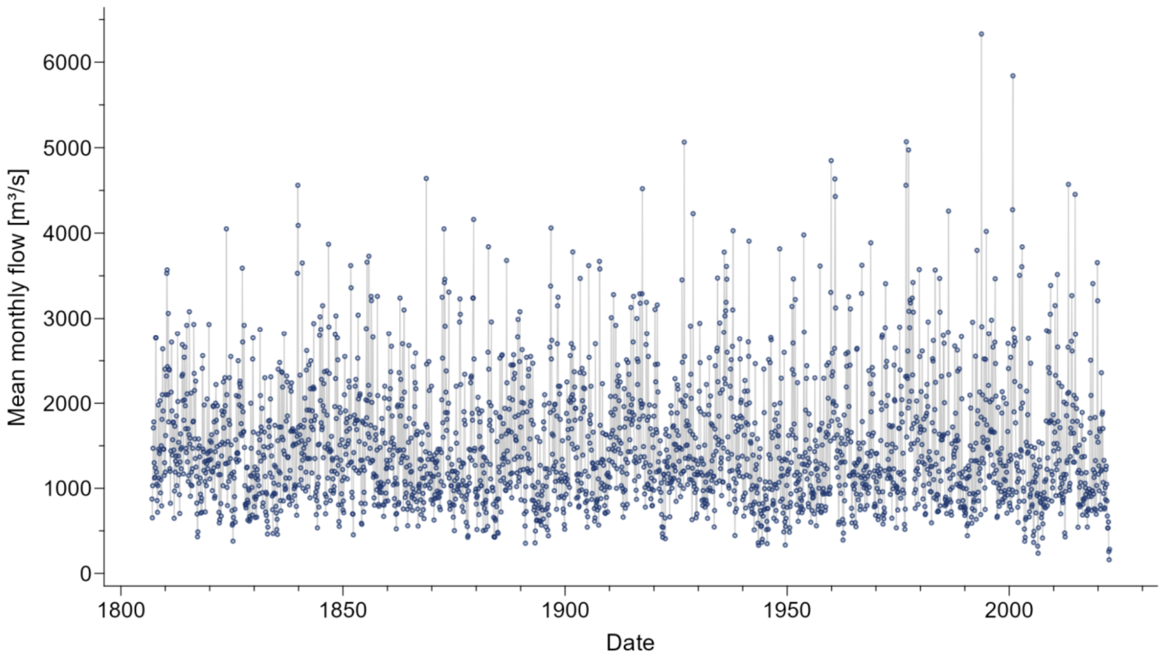

Fig. S1.
Mean monthly river flows of the Po River basin (Italy) at Pontelagoscuro from January 1807 to August 2022.

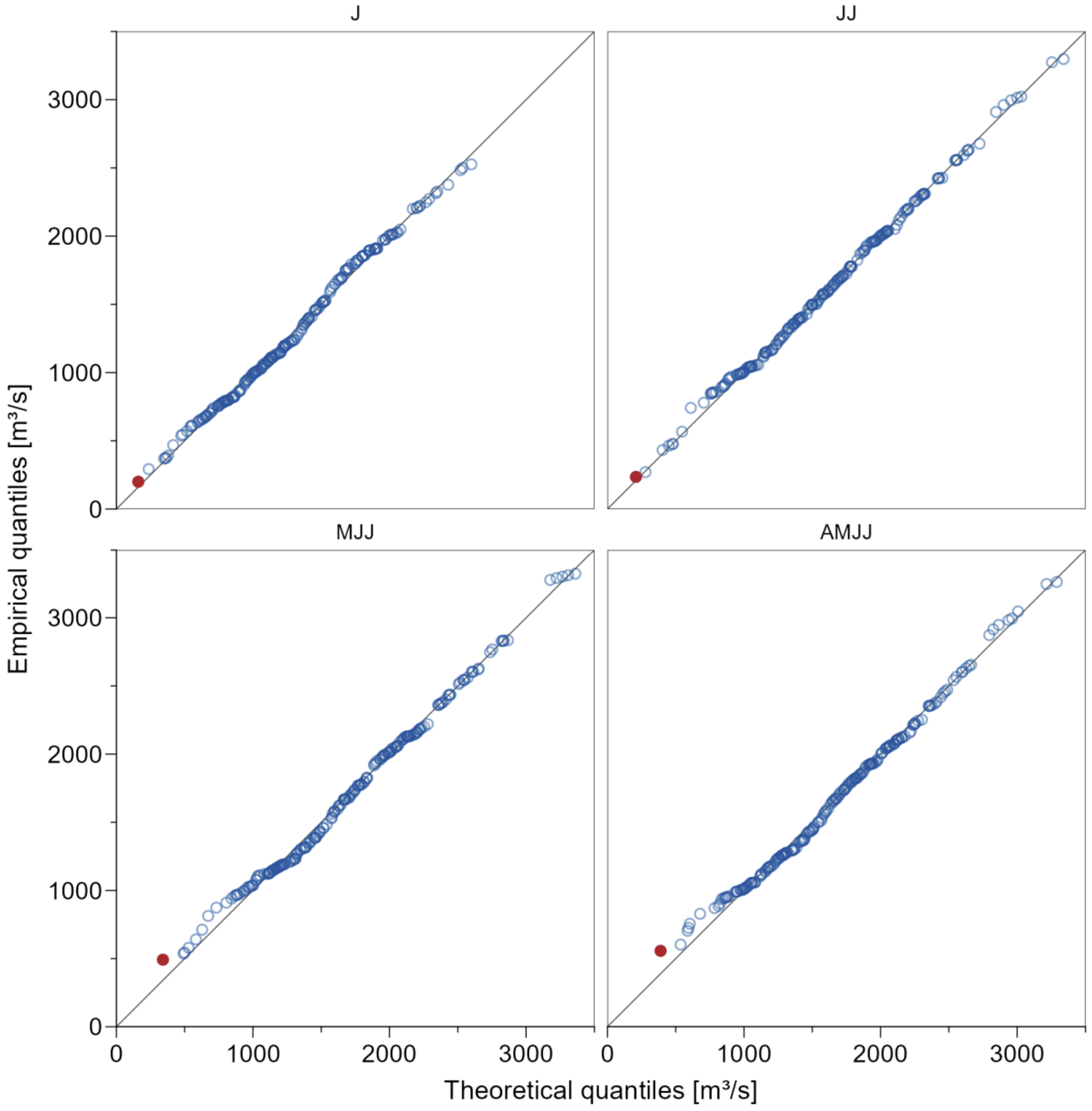

Fig. S2.

Quantile-quantile plots for the seasonal aggregation windows in Fig. 1 of the main text, July (J), June-July (JJ), May to July (MJJ), April to July (AMJJ). The red dots correspond to the year 2022.

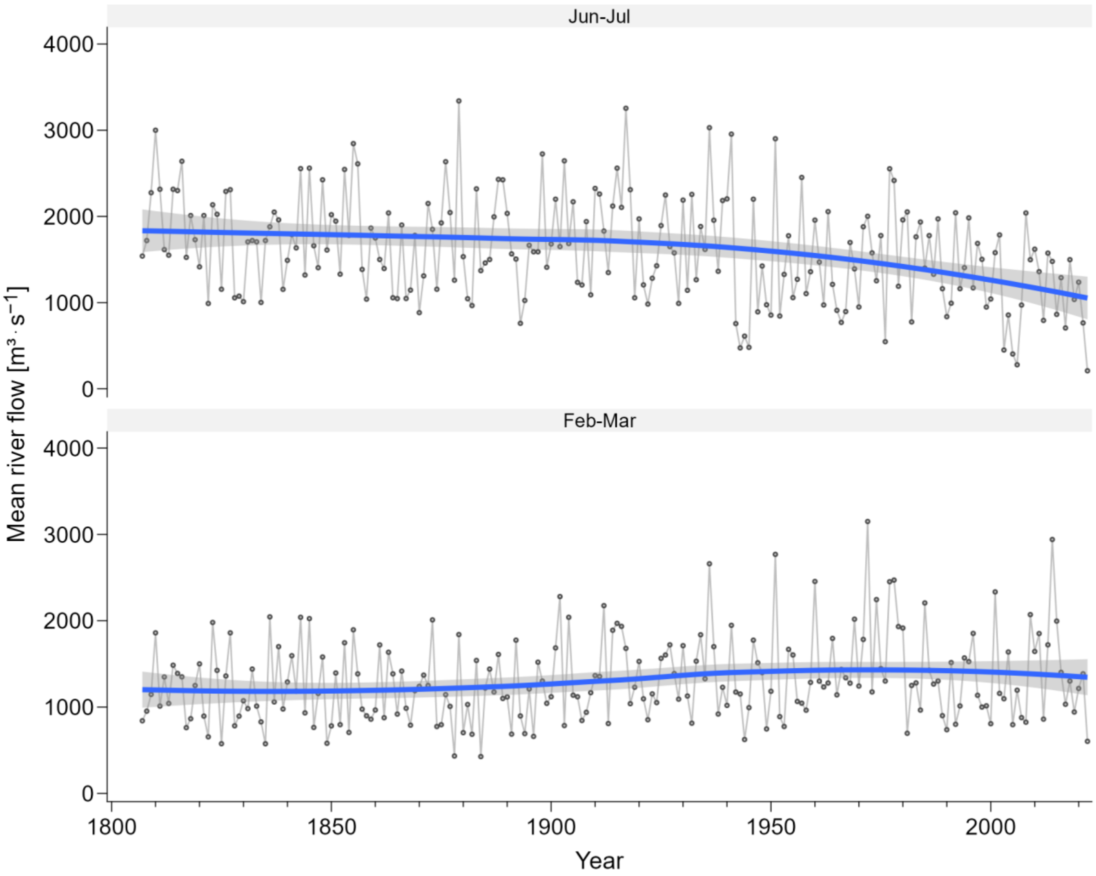

Fig. S3.

Seasonal Po River flows for bimonthly windows Feb-Mar and Jun-Jul. The blue line shows a lowess smoothing interpolation with polynomial degree 2 and span 1.

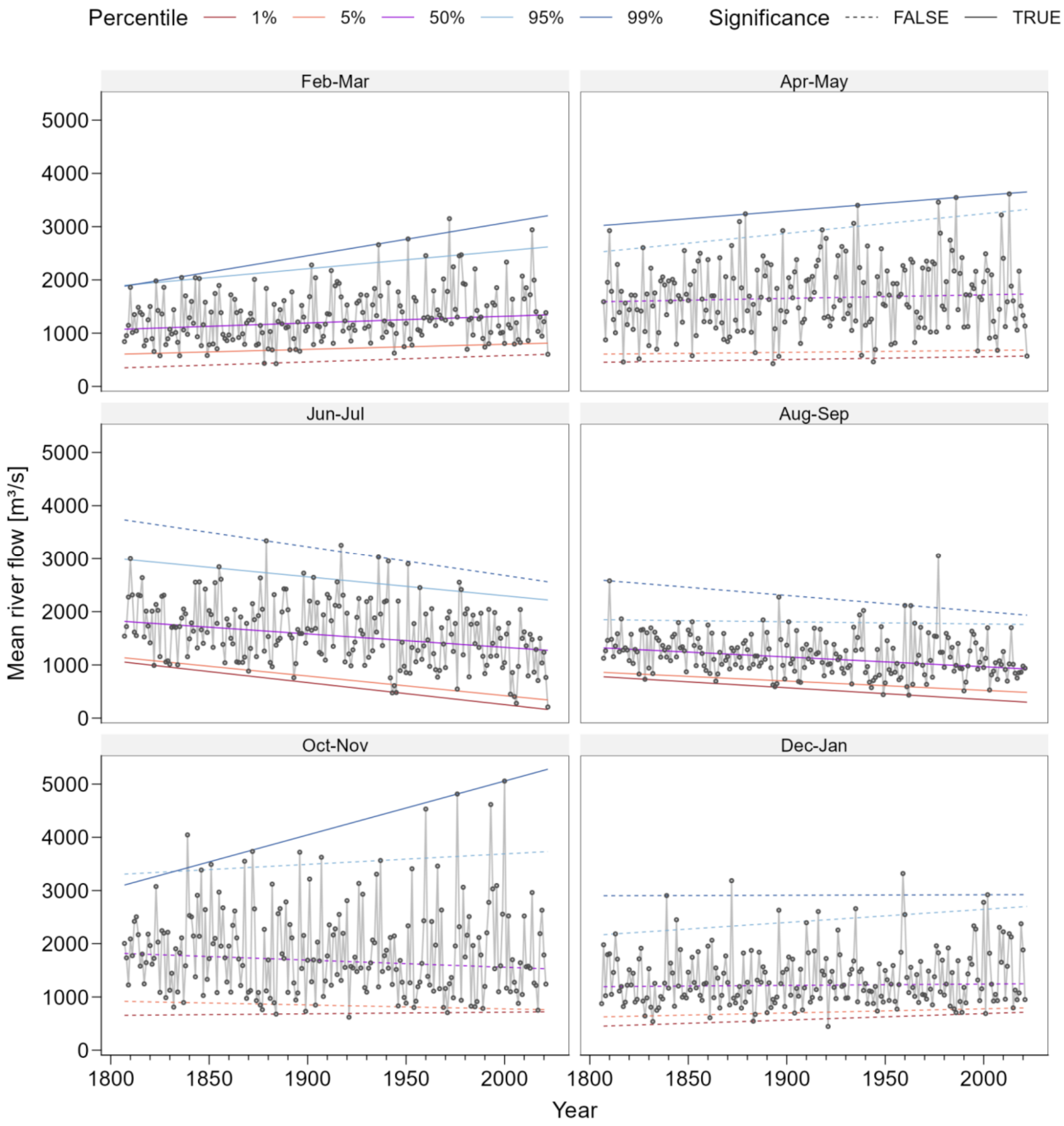

Fig. S4.

Seasonal Po River flows and quantile regression for bimonthly windows. Feb-Mar and Jun-Jul are shown in Fig. 2 of the main text.

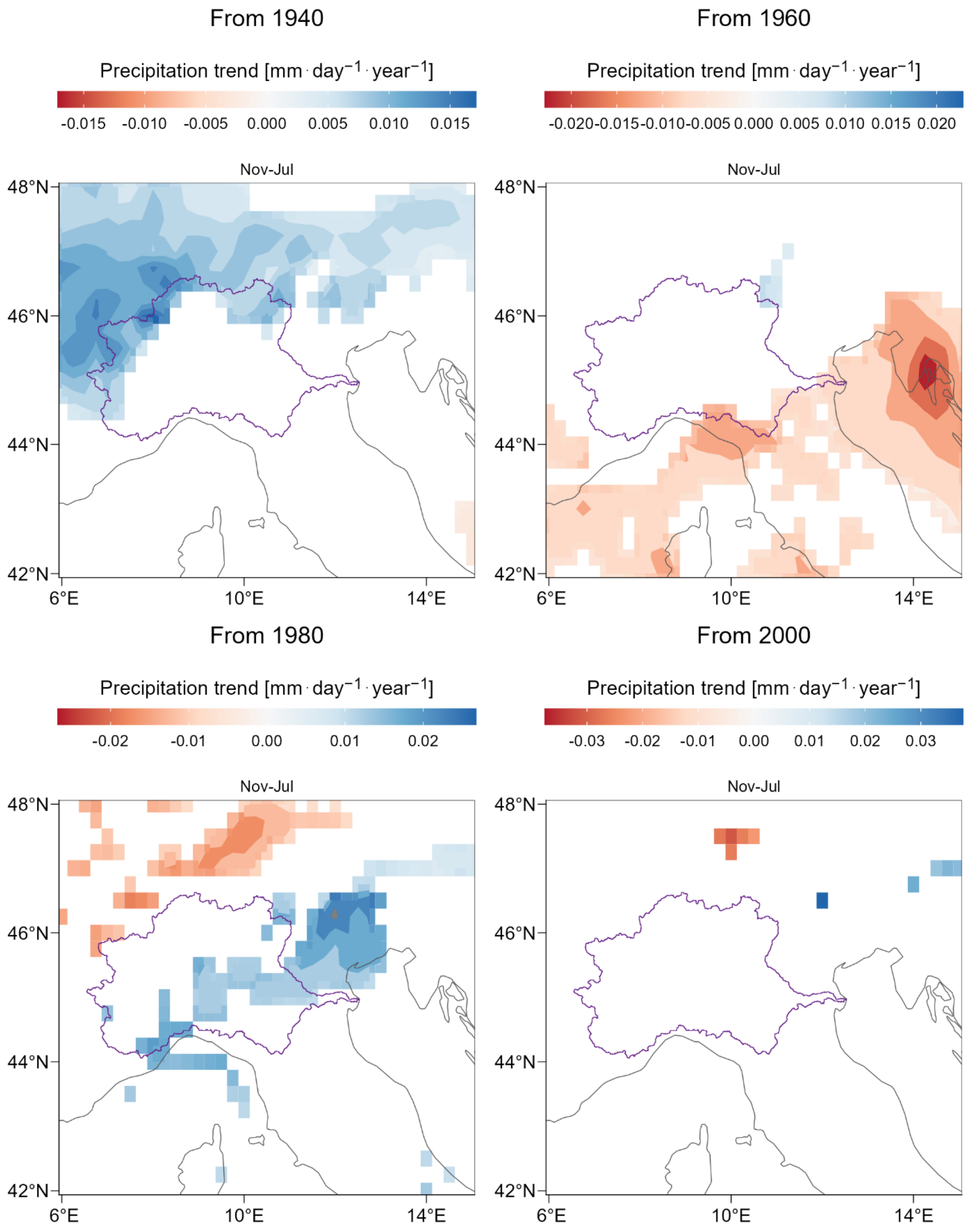

Fig. S5.

Change in cumulative November-July precipitation. Trend estimation for the period 1940-2022 over different time windows from ERA5 Reanalysis monthly data (*23*). Only significant trends ($p < 0.1$) are shown. No significant declining trends are found in the mean areal precipitation. The Po River basin boundary is shown in purple.

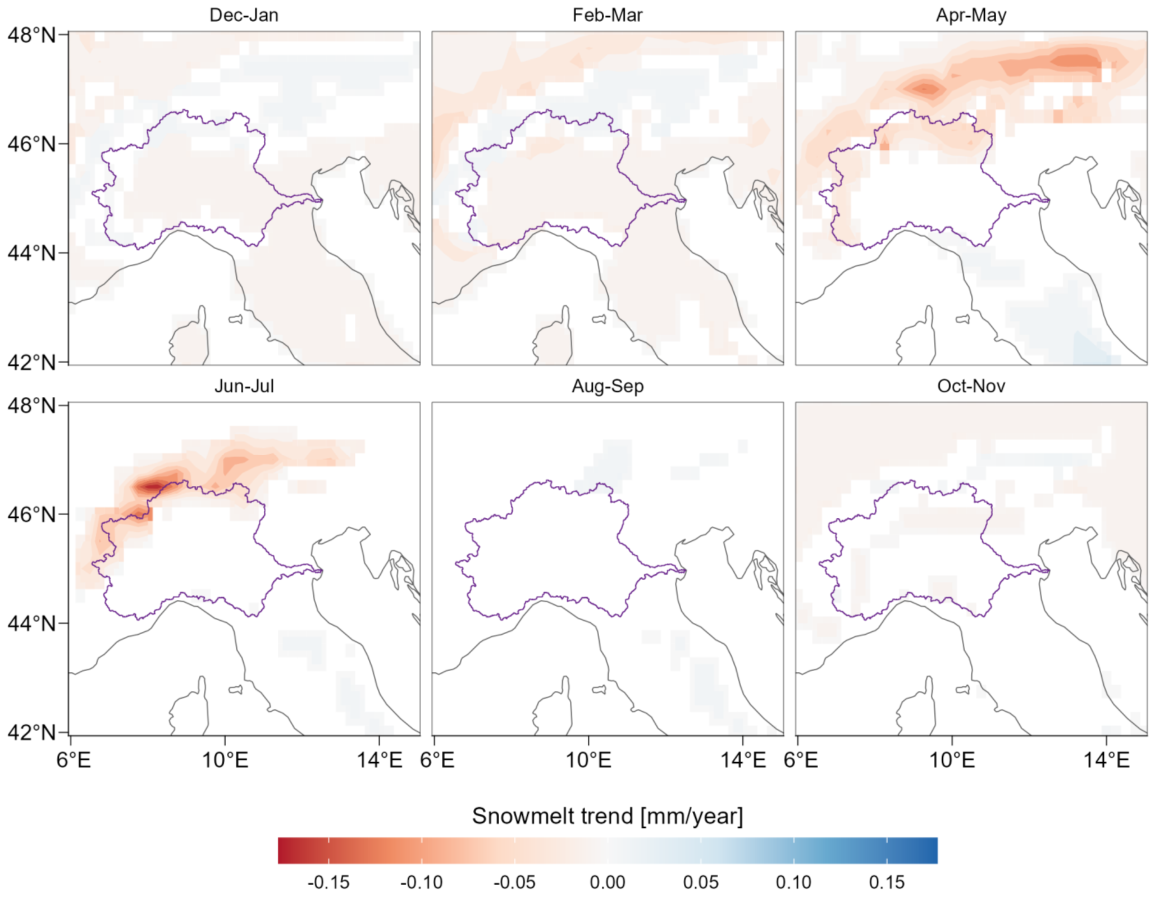

Fig. S6.
Change in Alpine snow regime. Snowmelt trend estimation for the period 1940-2022 from ERA5 Reanalysis bimonthly data (*23*). Only significant trends ($p < 0.1$) are shown. The Po River basin boundary is shown in purple.

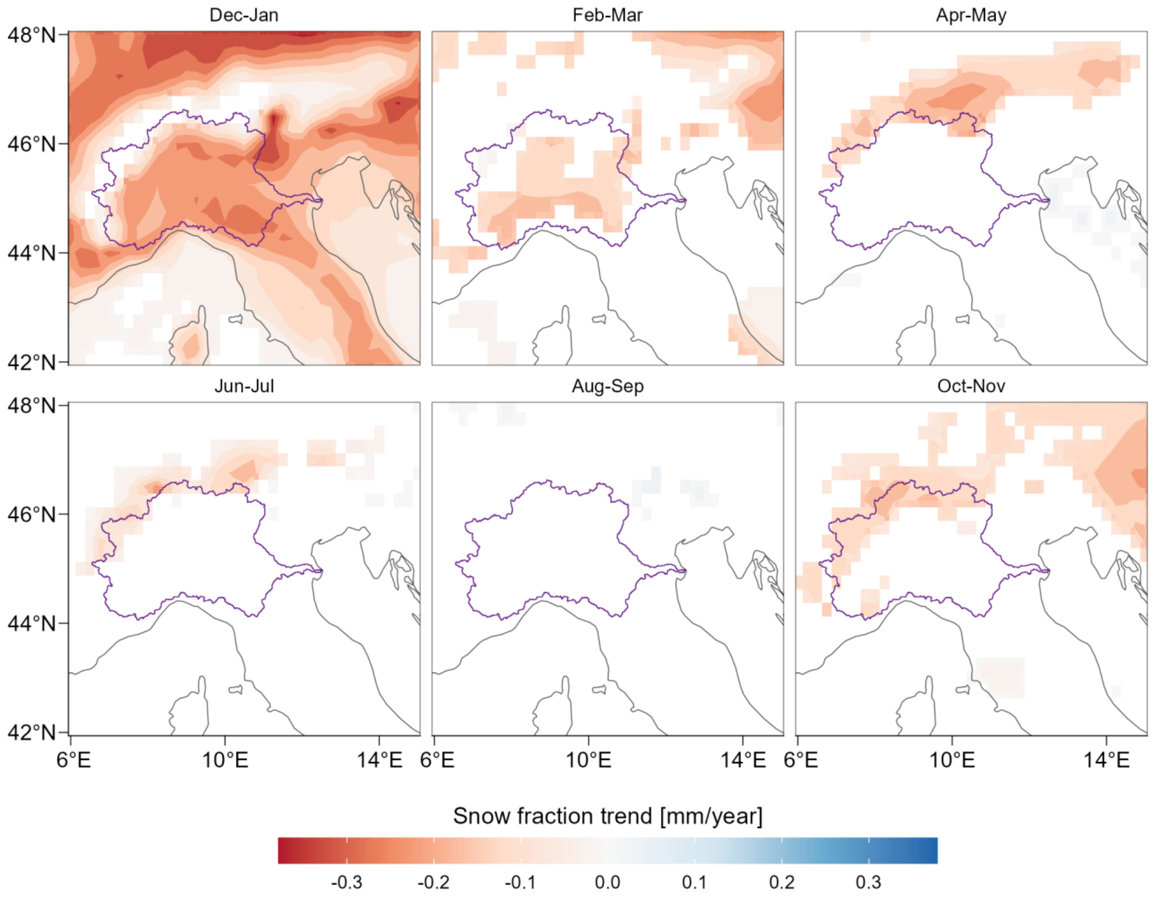

Fig. S7.
Change in Alpine snow regime. Snow fraction trend estimation for the period 1940-2022 from ERA5 Reanalysis bimonthly data (*23*). Snow fraction is calculated as the ratio between snowfall and total precipitation. Only significant trends ($p < 0.1$) are shown. The Po River basin boundary is shown in purple.

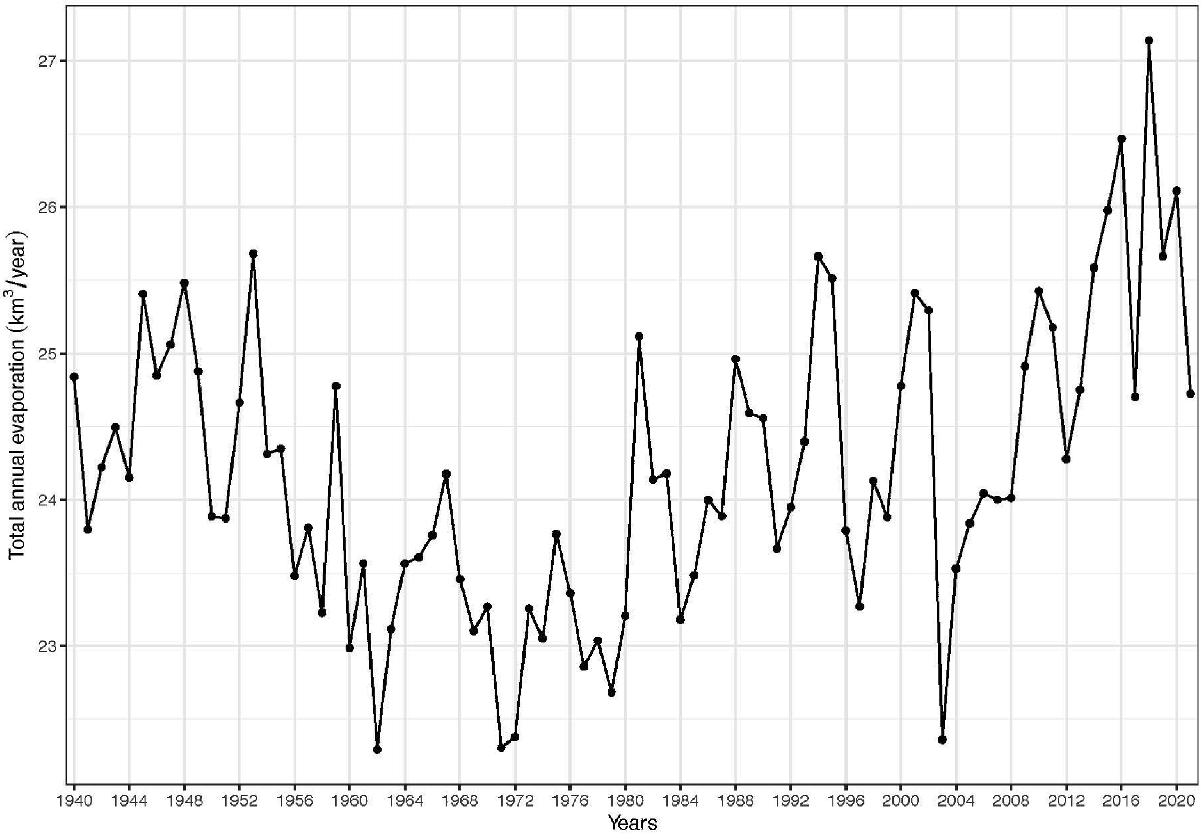

Fig. S8.

Change in total annual evaporation across the Po River basin. Data for the period 1940-2022 are retrieved from the ERA5 Reanalysis monthly data (*23*).

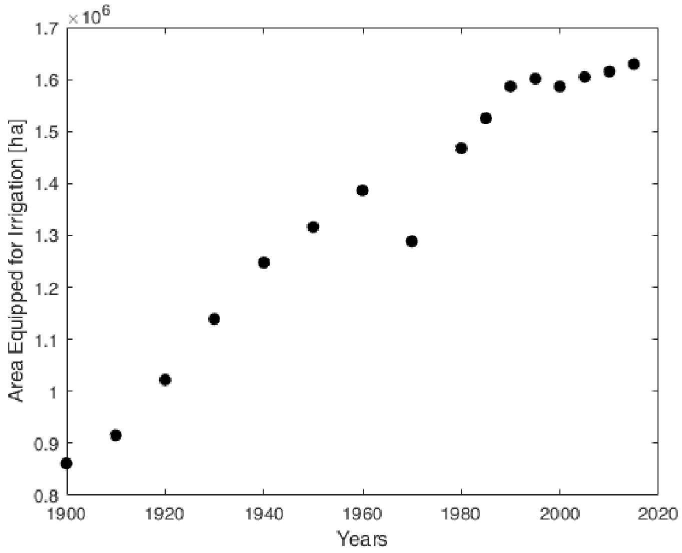

Fig. S9.
Change in Area Equipped for Irrigation across the Po River basin, retrieved from the Global Area Equipped for Irrigation Dataset 1900-2015 (*41*).

Table S1.
Return periods for the five worst drought years, based on the aggregation windows, as shown in Fig. 1 of the main text.

	Return period [year]			
**Year**	**J**	**JJ**	**MJJ**	**AMJJ**
2022	416.42	565.48	212.28	152.67
2006	143.93	243.26	65.38	37.57
2005	44.87	82.1	32.3	14.63
2003	40.3	59.95	40.62	25.89
1945	49.86	49.4	68.42	41.04

Table S2.
River flow trend estimation (from 1807 to 2022) for the seasons July (J), June-July (JJ), May to July (MJJ), April to July (AMJJ), February-March (FM), and whole Po River flow time series (in $m^3\ s^{-1}\ year^{-1}$), along with corresponding p-values in parentheses. (*) denotes *p*-values that are less than 0.01. Trends that are not statistically significant at the 10% level are greyed out.

	Slope [$m^3\ s^{-1}\ year^{-1}$]				
**Season**	**1%**	**5%**	**50%**	**95%**	**99%**
**J**	-3.66 (*)	-3.04 (*)	-3.67 (*)	-2.17 (0.14)	0.29 (0.85)
**JJ**	-4.14 (*)	-3.68 (*)	-2.53 (0.01)	-3.57 (0.07)	-5.44 (0.15)
**MJJ**	-3.83 (*)	-2.68 (*)	-2.42 (0.01)	-1.62 (0.44)	1.1 (0.68)
**AMJJ**	-2.27 (0.01)	-1.3 (0.16)	-1.06 (0.27)	0.38 (0.82)	-2.9 (0.32)
**FM**	1.18 (0.21)	0.96 (0.06)	1.26 (0.05)	3.35 (0.03)	6.16 (*)
**Full monthly time series**	-0.002 (0.07)	-0.001 (0.02)	-0.003 (*)	0.005 (0.04)	0.011 (0.03)

Table S3.

Trend estimation of monthly surface air temperature averaged over the Po River basin (in °C year^{-1}). (*) denotes *p*-values that are less than 0.01. Trends that are not statistically significant at the 10% level are greyed out.

	Slope [°C year^{-1}]	
**Month**	**HISTALP (1801-2014)**	**ERA5 (1940-2022)**
January	$9.8 \cdot 10^{-3}$ (*)	$5.3 \cdot 10^{-2}$ (*)
February	$4.3 \cdot 10^{-3}$ (0.05)	$4.1 \cdot 10^{-2}$ (*)
March	$5.8 \cdot 10^{-3}$ (*)	$3.2 \cdot 10^{-2}$ (*)
April	$2.0 \cdot 10^{-3}$ (0.18)	$2.2 \cdot 10^{-2}$ (*)
May	$1.7 \cdot 10^{-3}$ (0.33)	$3.0 \cdot 10^{-2}$ (*)
June	$2.5 \cdot 10^{-3}$ (0.10)	$3.9 \cdot 10^{-2}$ (*)
July	$3.6 \cdot 10^{-3}$ (0.01)	$3.1 \cdot 10^{-2}$ (*)
August	$3.6 \cdot 10^{-3}$ (0.01)	$3.0 \cdot 10^{-2}$ (*)
September	$3.5 \cdot 10^{-3}$ (0.02)	$1.0 \cdot 10^{-2}$ (0.10)
October	$3.9 \cdot 10^{-3}$ (0.01)	$2.4 \cdot 10^{-2}$ (*)
November	$5.9 \cdot 10^{-3}$ (*)	$3.5 \cdot 10^{-2}$ (*)
December	$5.9 \cdot 10^{-3}$ (*)	$3.7 \cdot 10^{-2}$ (*)

Table S4.

Sen's slope estimation of monthly sectoral water withdrawals aggregated over the Po River basin (in m^3 $month^{-1}$ $year^{-1}$), retrieved from the global gridded monthly sectoral water use dataset for 1971-2010: v2 (*40*), along with corresponding *p*-values in parentheses. (*) denotes *p*-values that are less than 0.01. Trends that are not statistically significant at the 10% level are greyed out. The relative distribution (as %) of sectoral water withdrawals is also shown.

	**Sen's slope [m^3 $month^{-1}$ $year^{-1}$]**			
**Sectoral water use**	**J**	**JJ**	**MJJ**	**AMJJ**
Domestic (~10%)	$1.87\cdot10^6$ (0.03)	$3.89\cdot10^6$ (0.03)	$5.57\cdot10^6$ (0.03)	$7.25\cdot10^6$ (0.04)
Electricity (~7%)	$4.43\cdot10^6$ (*)	$8.69\cdot10^6$ (*)	$1.23\cdot10^7$ (*)	$1.62\cdot10^7$ (*)
Irrigation (~75%)				
H08	$-5.11\cdot10^7$ (0.07)	$-4.07\cdot10^7$ (0.29)	$-2.44\cdot10^7$ (0.58)	$-2.69\cdot10^7$ (0.48)
LPJmL	$-2.03\cdot10^7$ (0.13)	$-1.07\cdot10^7$ (0.79)	$4.53\cdot10^6$ (0.95)	$8.43\cdot10^6$ (0.84)
PCR-GLOBWB	$-3.62\cdot10^6$ (0.77)	$-1.61\cdot10^7$ (0.30)	$-2.27\cdot10^7$ (0.32)	$-2.46\cdot10^7$ (0.29)
WaterGAP	$-6.45\cdot10^6$ (0.60)	$7.04\cdot10^6$ (0.68)	$1.01\cdot10^7$ (0.62)	$9.08\cdot10^6$ (0.62)
Livestock (~0.3%)	$-1.69\cdot10^4$ (0.68)	$-3.31\cdot10^4$ (0.68)	$-5\cdot10^4$ (0.68)	$-6.64\cdot10^4$ (0.68)
Manufacturing (~7%)	$-2.85\cdot10^6$ (*)	$-5.61\cdot10^6$ (*)	$-8.47\cdot10^6$ (*)	$-1.12\cdot10^7$ (*)
Mining (~0.7%)	$-2.81\cdot10^5$ (*)	$-5.53\cdot10^5$ (*)	$-8.34\cdot10^5$ (*)	$-1.11\cdot10^6$ (*)
